# Supplementary material for: Drivers of associations between daytime-nighttime compound temperature extremes and mortality in China
Source: Commun Med (Lond). 2024 Jun 27;4:125. doi: 10.1038/s43856-024-00557-0 (PMC11211425; doi:10.1038/s43856-024-00557-0)
Supplement: Supplementary file 2 — Supplemental information [file 43856_2024_557_MOESM2_ESM.pdf]

Supplementary Information

Drivers of associations between daytime-nighttime compound temperature extremes and mortality in China

Jun Yang[1,†], Maigeng Zhou[2,†], Cui Guo[3],Sui Zhu[4], Mohammad Javad Zare Sakhvidi[5], Weeberb J.Requia[6], Qinghua Sun[7], Shilu Tong[8,9,10], Mengmeng Li[11], Qiyong Liu[12]

[1] School of Public Health, Guangzhou Medical University, Guangzhou, 511436, China
[2] National Center for Chronic and Noncommunicable Disease Control and Prevention, Beijing 100050, China
[3] Department of Urban Planning and Design, Faculty of Architecture, The University of Hong Kong, Hong Kong SAR, China
[4] Department of Public Health and Preventive Medicine, School of Medicine, Jinan University, Guangzhou, 510080, China
[5] Department of Occupational Health, School of Public Health, Shahid Sadoughi University of Medical Sciences, Yazd, Iran
[6] School of Public Policy and Government, Fundação Getúlio Vargas, Brasília, Distrito Federal, Brazil
[7] School of Public Health, Zhejiang Chinese Medical University, Hangzhou, 310053, China
[8] Shanghai Children's Medical Center, Shanghai Jiao Tong University, Shanghai 200127, China
[9] School of Public Health and Institute of Environment and Population Health, Anhui Medical University, Hefei, China
[10] School of Public Health and Institute of Health and Biomedical Innovation, Queensland University of Technology, Brisbane, Australia.
[11] State Key Laboratory of Oncology in South China, Guangdong Provincial Clinical Research Center for Cancer, Sun Yat-sen University Cancer Center, Guangzhou, China
[12] National Key Laboratory of Intelligent Tracking and Forecasting for Infectious Diseases, National Institute for Communicable Disease Control and Prevention, Chinese Center for Disease Control and Prevention, Beijing, China

[†] Co-first authors.

Correspondence to: Jun Yang (yangjun_eci@jnu.edu.cn); Qiyong Liu (liuqiyong@icdc.cn).

Supplementary Method

Identification of threshold for heat wave and cold spell based on mortality residuals

First, in order to eliminate the long-term and seasonal trend of daily death series, the autoregressive integrated moving average (ARIMA) was used for daily counts of non-accidental mortality[1,2]. Then, we separately incorporated the mortality residuals obtained from ARIMA with daily maximum temperature and minimum temperature at 1°C. Threshold would be the temperature above (for heat wave) or below (for cold wave) which mortality residuals are consistently and significantly increased. The data analyses of heat wave were restricted to the hot season (May to September) and cold spell to the cold season (November to March)

References

1. Martinez GS, Diaz J, Hooyberghs H, et al. Heat and health in Antwerp under climate change: Projected impacts and implications for prevention. *Environment International* 2018; **111**: 135-43.
2. Box GE, Jenkins GM, Reinsel GC, Ljung GM. Time series analysis: forecasting and control: John Wiley & Sons; 2015.

Supplementary Table 1. Descriptive statistics of annual amounts of heat wave and cold spell during 2007-2013 in 161 Chinese districts/counties.

Variable	Mean	SD	Minimum	P25	P50	P75	Maximum
Heat wave							
Daytime only	3.9	2.7	0	1	5	6	7
Nighttime only	4.2	3	0	1	5	6	9
Compound	2.3	2	0	0	3	3	5
Cold spell							
Daytime only	3.5	2.5	0	2	3	5	9
Nighttime only	3.6	1.5	1	2	3	5	6
Compound	3.1	2.2	0	2	3	5	8

Note. SD denotes standard deviation.

Supplementary Table 2. Average values of Akaike's Information Criteria for quasi-Poisson (Q-AIC) for 18 heat wave definitions in 31 Chinese provinces.

Province	P75_2d	P75_3d	P77.5_2d	P77.5_3d	P80_2d	P80_3d	P82.5_2d	P82.5_3d	P85_2d	P85_3d	P87.5_2d	P87.5_3d	P90_2d	P90_3d	P92.5_2d	P92.5_3d	P95_2d	P95_3d
Anhui	2982.2	2982.16	2982.45	2982.54	2982.43	2982.6	2982.42	2982.57	2982.3	2982.28	2982.21	2982.54	2982.35	2982.44	2982.14	2982.23	2981.89	2982.11
Beijing	4111.7	4111.56	4111.98	4112.11	4111.74	4112.21	4111.2	4111.83	4111.45	4111.86	4111.5	4111.81	4111.54	4111.72	4112.07	4112.48	4111.86	4112.44
Chongqing	3787.93	3787.89	3787.84	3787.55	3787.73	3787.05	3787.43	3786.81	3787.5	3786.88	3787.58	3787.35	3787.45	3787.63	3787.38	3787.61	3787.76	3787.45
Fujian	3275.41	3275.3	3275.27	3275.4	3275.11	3275.49	3275.31	3275.13	3275.29	3275.07	3275.51	3275.42	3275.7	3275.87	3275.73	3276.15	3276.18	3276.27
Gansu	3057.31	3057.6	3057.48	3057.22	3057.29	3057.2	3057.15	3057.33	3057.28	3057.55	3057.47	3057.56	3057.61	3057.58	3057.6	3057.55	3057.68	3057.68
Guangdong	4065.23	4065.13	4065.3	4065.15	4065.19	4065.2	4064.82	4065.08	4064.86	4065.11	4065.01	4065.05	4064.81	4065	4064.73	4064.36	4064.66	4063.88
Guangxi	3431.09	3431.18	3431.01	3430.67	3430.86	3431.01	3431.4	3431.61	3431.32	3431.38	3431.58	3431.24	3431.66	3431.36	3431.61	3431.4	3431.31	3431.53
Guizhou	2917.67	2917.56	2917.98	2917.8	2917.79	2917.73	2917.61	2917.41	2917.82	2917.83	2918.02	2918.02	2917.95	2918	2917.85	2917.95	2917.71	2917.85
Hainan	2754.64	2753.78	2753.7	2752.83	2753.71	2752.42	2753.8	2753.29	2754.14	2754.16	2753.66	2754.03	2754.56	2754.41	2754.42	2754.46	2753.8	2753.84
Hebei	3331.41	3331.07	3331.65	3331.59	3331.57	3331.92	3332.96	3332.4	3332.63	3332.9	3332.47	3332.45	3332.87	3332.16	3332.68	3332.6	3332.34	3332.9
Heilongjiang	3256.44	3256.36	3256.56	3256.5	3256.69	3256.6	3256.84	3256.57	3256.83	3256.53	3256.82	3256.48	3256.6	3256.39	3256.73	3255.94	3256.2	3256.51
Henan	4329.52	4329.71	4329.49	4329.19	4329.68	4329.04	4329.94	4329.53	4329.88	4329.4	4330.1	4329.96	4329.93	4329.78	4329.87	4329.55	4330.43	4330.16
Hubei	3577.26	3576.82	3577.2	3576.87	3577.09	3576.57	3576.78	3576.28	3576.96	3576.82	3576.76	3576.56	3576.98	3577.16	3577.46	3577.23	3577.08	3577.27
Hunan	3838.99	3839.29	3839.24	3839.46	3839.62	3839.51	3839.79	3839.57	3839.58	3839.36	3839.56	3839.73	3839.86	3839.83	3839.72	3839.35	3839.47	3839.57
Inner Mongolia	2956.25	2956.07	2956.05	2956.06	2955.84	2955.89	2955.82	2955.77	2955.58	2955.66	2955.92	2955.53	2955.99	2955.96	2955.91	2955.62	2955.62	2955.78
Jiangsu	3657.32	3657.51	3657.49	3657.55	3657.45	3657.75	3657.28	3657.4	3656.78	3656.92	3656.89	3657.21	3656.82	3656.69	3656.95	3656.69	3657	3657.33
Jiangxi	2792.34	2792.32	2792.38	2792.5	2792.11	2792.37	2792.17	2792.38	2792.5	2792.48	2792.66	2792.35	2792.7	2792.3	2792.99	2792.68	2792.6	2792.57
Jilin	3142.92	3142.67	3143.09	3142.76	3143.18	3142.91	3142.92	3143.11	3142.95	3143.13	3142.77	3142.76	3142.58	3142.69	3142.69	3142.86	3142.73	3143
Liaoning	3791.29	3791.15	3791.13	3790.78	3791.04	3791.04	3790.95	3790.98	3790.88	3790.85	3790.8	3790.93	3790.9	3791.04	3790.94	3791.23	3790.96	3791.23
Ningxia	3294.09	3294.3	3293.98	3294.25	3294	3294.4	3294.36	3294.61	3294.54	3294.49	3294.31	3294.21	3294.01	3293.53	3293.91	3293.18	3293.41	3293.3
Qinghai	2201.58	2202.11	2201.67	2202.07	2201.88	2201.99	2201.95	2202.02	2201.58	2202	2202.01	2202.03	2201.7	2201.76	2201.79	2201.44	2201.95	2201.63
Shaanxi	2805.93	2805.99	2806.04	2805.96	2805.93	2805.69	2805.74	2805.8	2805.72	2805.82	2805.77	2805.77	2805.75	2805.79	2805.88	2805.71	2805.85	2805.93
Shandong	4043.53	4043.91	4043.5	4043.93	4043.5	4043.35	4042.81	4042.8	4042.74	4042.28	4042.21	4042.19	4042.06	4042.16	4041.81	4042.27	4042.1	4042.43
Shanghai	2974.81	2974.7	2974.84	2975.19	2975.2	2975.48	2975.31	2975.66	2975.49	2975.83	2975.18	2975.48	2974.8	2975.47	2974.92	2974.64	2974.8	2975.11
Shanxi	3276.74	3276.75	3276.72	3276.79	3276.97	3276.68	3277.07	3276.92	3276.83	3277.06	3277.3	3277.04	3277.38	3277.3	3277.34	3277.02	3276.94	3276.77
Sichuan	3668.28	3667.97	3668.31	3668.06	3668.1	3667.5	3667.59	3667.14	3667.78	3667.78	3667.87	3667.82	3668.2	3668.16	3668	3667.74	3668.17	3668.09
Tianjin	3750	3750.5	3750.2	3750.52	3750.47	3750.58	3750.6	3750.68	3750.85	3750.63	3750.82	3750.71	3750.23	3750.6	3750.65	3750.42	3750.68	3749.93
Tibet	1358.85	1361.92	1358.74	1362.84	1359.52	1364.45	1359.42	1361.22	1359.16	1359.29	1359.02	1359.5	1358.9	1359.23	1359.1	1359.46	1359.55	1360.92
Xinjiang	3114.3	3114.33	3114.3	3114.33	3113.99	3114.33	3114.39	3114.26	3114.68	3114.17	3114.65	3114.34	3114.47	3114.08	3114.45	3114.51	3114.43	3114.81
Yunnan	3443.1	3443.11	3442.99	3443.11	3443.01	3443.03	3442.88	3442.92	3442.97	3443	3443.04	3443.13	3442.88	3443.25	3442.92	3443.33	3442.91	3443.22
Zhejiang	3289.89	3289.68	3289.69	3289.8	3289.7	3289.94	3290.06	3290.11	3289.88	3289.8	3289.8	3289.89	3289.77	3289.6	3289.61	3289.55	3288.8	3289.3

Note. "P" denotes percentile and "d" denotes the duration.

Supplementary Table 3. Average values of Akaike's Information Criteria for quasi-Poisson (Q-AIC) for 18 cold spell definitions in 31 Chinese provinces.

Province	P25_2d	P25_3d	P22.5_2d	P22.5_3d	P20_2d	P20_3d	P17.5_2d	P17.5_3d	P15_2d	P15_3d	P12.5_2d	P12.5_3d	P10_2d	P10_3d	P7.5_2d	P7.5_3d	P5_2d	P5_3d
Anhui	3237.08	3236.92	3236.96	3237.13	3236.91	3236.78	3237.03	3237.08	3237.1	3237.34	3237.28	3237.43	3237.7	3237.99	3237.44	3237.78	3237.8	3238.2
Beijing	4390.08	4389.99	4390.07	4389.85	4389.85	4390.23	4390.15	4390.59	4390.59	4390.4	4390.38	4390.03	4390.32	4390.15	4389.96	4389.96	4390.19	4390.8
Chongqing	4019.94	4019.51	4020.24	4020.29	4020.49	4020.77	4020.07	4020.76	4020.53	4020.88	4021.05	4021.42	4021.52	4021.47	4021.2	4021.13	4021.07	4020.81
Fujian	3461.03	3461.05	3460.8	3460.75	3460.97	3461.01	3460.51	3461.06	3460.89	3461.28	3460.53	3461.62	3460.17	3461.9	3460.59	3461.87	3461.81	3463.17
Gansu	3336.89	3336.77	3336.86	3336.87	3336.76	3336.96	3336.32	3336.89	3336.63	3337.02	3336.82	3336.86	3337.12	3337.2	3336.94	3337.16	3337.52	3337.7
Guangdong	4265.23	4265.91	4264.96	4265.1	4264.84	4264.31	4263.68	4263.45	4262.68	4262.81	4263.53	4263.7	4262.71	4264.51	4263.66	4265.25	4264.09	4265.65
Guangxi	3676.96	3676.73	3677.23	3677.24	3677.32	3677.59	3677.24	3677.33	3677.62	3677.49	3677.79	3677.39	3677.58	3676.5	3677.4	3677.48	3676.99	3677.5
Guizhou	3091.17	3090.95	3090.8	3090.93	3090.95	3091.23	3090.93	3091.29	3091.18	3091.5	3091.45	3091.68	3091.41	3091.42	3091.39	3091.26	3091.55	3091.39
Hainan	2817.93	2817.9	2817.78	2817.76	2817.31	2817.43	2817.57	2817.26	2817	2817.13	2817.18	2816.67	2816.6	2816.93	2817.14	2817.12	2816.91	2816.57
Hebei	3769.99	3769.99	3769.95	3769.05	3769.54	3769.38	3770.23	3769.28	3770.36	3769.82	3769.45	3770.21	3770	3769.75	3768.72	3766.93	3766.93	3766.35
Heilongjiang	3423.73	3424.3	3423.52	3423.39	3423.49	3423.79	3423.23	3423.61	3423.7	3423.75	3423.43	3423.67	3423.36	3423.59	3423.76	3423.44	3423.55	3423.51
Henan	4809.97	4809.7	4810.96	4811.25	4811.51	4811.51	4811.25	4811.99	4811.85	4812.16	4812.3	4812.67	4811.99	4812.34	4812.74	4813.61	4813.61	4813.68
Hubei	3925.58	3925.33	3925.39	3925.51	3925.19	3925.66	3925.85	3926.26	3926.14	3926.19	3926.02	3925.87	3925.84	3925.76	3925.57	3925.84	3925.62	3925.87
Hunan	4179.97	4180.7	4178.3	4179.52	4179.29	4180.12	4180.4	4180.38	4180.39	4180.33	4179.98	4180.06	4179.36	4178.97	4180.09	4179.55	4180.55	4180.37
Inner Mongolia	3090.19	3089.91	3089.34	3089.69	3089.54	3089.25	3089.87	3089.74	3089.82	3089.76	3089.69	3090.26	3089.34	3089.76	3089.5	3089.6	3089.54	3090
Jiangsu	3949.65	3949.64	3949.25	3949.09	3949.27	3949.79	3948.98	3949.59	3949.33	3949.43	3949.43	3949.77	3949.96	3949.49	3950.22	3949.91	3950.66	3950.82
Jiangxi	3073.47	3073.48	3073.84	3074.23	3074.02	3073.89	3073.46	3073.79	3073.4	3073.8	3073.37	3072.95	3074.23	3074.05	3073.54	3074.32	3073.68	3075.19
Jilin	3163.33	3163.38	3163.37	3163.38	3163.2	3163.41	3163.29	3163.15	3163.34	3163.57	3163.46	3163.34	3163.67	3163.61	3163.86	3163.41	3163.73	3163.45
Liaoning	4040.02	4040.13	4039.95	4040.2	4040.06	4040.41	4040.08	4040.17	4039.83	4040.09	4039.96	4040.13	4039.56	4039.93	4039.29	4040.02	4039.48	4039.41
Ningxia	3537.05	3536.29	3536.44	3536.37	3536.65	3536.59	3536.42	3536.46	3537.13	3537.16	3536.83	3537.22	3536.58	3537.24	3537.15	3537.09	3536.5	3536.58
Qinghai	2399.56	2399.65	2399.56	2399.49	2399.64	2399.64	2399.52	2399.54	2399.59	2399.52	2399.81	2399.65	2399.83	2399.79	2399.87	2399.81	2399.65	2399.85
Shaanxi	3215.52	3215.55	3215.2	3215.39	3215.41	3215.45	3215.24	3215.46	3215.36	3215.69	3215.23	3215.59	3215.06	3215.44	3215.13	3215.67	3215.7	3215.89
Shandong	4410.97	4411.31	4411.81	4412.23	4411.52	4412.05	4411.57	4412.12	4411.02	4412.06	4411.45	4412.23	4411.77	4412.06	4411.72	4412.4	4412.34	4412.72
Shanghai	3217.68	3217.45	3217.22	3217.03	3216.9	3216.84	3216.8	3217.49	3217.07	3217.84	3217.67	3218.43	3218.11	3218.05	3218.35	3218.41	3218.1	3218.67
Shanxi	3552.67	3553.57	3553.5	3554.55	3553.56	3554.89	3554.52	3556.57	3556.82	3558.27	3557.57	3558.91	3557.68	3558.75	3558.22	3559.07	3558.11	3559.66
Sichuan	3829.05	3829.26	3829.18	3829.51	3829.11	3829.61	3829.94	3829.99	3829.99	3829.96	3830.02	3829.95	3830.16	3830.48	3829.96	3830.35	3829.87	3830.27
Tianjin	4052.65	4052.45	4051.84	4051.45	4052.21	4051.77	4051.67	4052	4051.01	4050.84	4051.19	4052.04	4051.99	4052.89	4052.28	4053.64	4051.88	4053.65
Tibet	1445.15	1445.23	1445.09	1445.16	1445.44	1445.1	1436.51	1445.2	1445.34	1445.48	1445.15	1445.19	1445.46	1445.62	1445.47	1445.53	1445.28	1445.3
Xinjiang	3413.46	3413.78	3415.49	3415.61	3415.74	3415.84	3415.7	3415.56	3415.67	3415.46	3415.88	3415.65	3415.45	3415.55	3414.74	3414.64	3414.76	3415.52
Yunnan	3597.24	3597.35	3597.2	3597.15	3597.18	3596.99	3596.95	3597.06	3596.96	3597.33	3597.21	3597.47	3597.05	3597.06	3597.09	3597.4	3597.59	3597.86
Zhejiang	3623.83	3623.93	3624.09	3624.16	3623.83	3624.25	3623.93	3624.33	3623.81	3624.33	3623.84	3624.51	3624.68	3625	3625.22	3625.35	3625.33	3625.76

Note. "P" denotes percentile and "d" denotes the duration.

Supplementary Table 4. Relative risk ratios (RRR) for effect estimates of temperature extremes between low and high levels of relative humidity.

Temperature extreme	Subcategory	Relative humidity	
		Low level	High level
Heat wave	Daytime only	1	1.019 (0.978, 1.062)
	Nighttime only	1	1.011 (0.980, 1.043)
	Compound	1	1.029 (0.995, 1.064)
Cold spell	Daytime only	1	0.922 (0.820, 1.037)
	Nighttime only	1	0.976 (0.873, 1.091)
	Compound	1	0.964 (0.910, 1.021)

Supplementary Table 5. Percentage change in mortality risk during heat wave at daytime only, nighttime only and compound across lag 0-1 days by cause, individual characteristics, and regions.

Variable	Daytime only	Nighttime only	Compound
Cause			
Non-accidental mortality	2.82 (1.21, 4.46)	1.16 (-0.75, 3.10)	8.86 (6.82, 10.94)
Cardiovascular mortality	3.72 (1.27, 6.23)	1.06 (-1.51, 3.69)	12.37 (9.20, 15.63)
IHD mortality	6.95 (3.24, 10.80)	2.32 (-1.44, 6.24)	13.44 (8.50, 18.60)
Stroke mortality	2.41 (-0.58, 5.50)	0.48 (-2.60, 3.66)	11.23 (7.79, 14.79)
Respiratory mortality	2.79 (-0.97, 6.70)	2.64 (-0.93, 6.33)	10.63 (6.54, 14.87)
COPD mortality	2.98 (-1.03, 7.16)	1.24 (-2.88, 5.53)	8.98 (4.42, 13.73)
Gender			
Male	2.06 (0.25, 3.89)	0.76 (-1.39, 2.95)	7.25 (5.20, 9.34)
Female	4.37 (1.99, 6.81)	1.81 (-0.64, 4.33)	10.84 (7.79, 13.98)
Age (years)			
0-74	1.86 (-0.06, 3.81)	1.35 (-0.74, 3.48)	5.25 (3.33, 7.21)
≥75	4.62 (2.08, 7.23)	1.07 (-1.35, 3.54)	12.70 (9.49, 16.00)
Educational level			
Illiterate	4.14 (1.59, 6.75)	0.43 (-1.95, 2.87)	13.50 (9.96, 17.15)
Primary school or higher	2.97 (1.09, 4.88)	1.79 (-0.68, 4.33)	6.73 (4.81, 8.69)
Region			
Urban	3.59 (0.81, 6.45)	1.25 (-1.82, 4.42)	11.11 (8.37, 13.92)
Rural	2.38 (0.41, 4.39)	1.10 (-1.32, 3.58)	7.29 (4.47, 10.19)
Climate			
Mid-temperature zone	5.60 (1.76, 9.58)	4.04 (-1.24, 9.61)	6.88 (1.95, 12.06)
Warm temperature zone	3.25 (0.68, 5.89)	3.02 (-0.14, 6.27)	9.79 (4.92, 14.89)
Qinghai-Tibet alpine zone	-1.39 (-7.06, 4.62)	2.29 (-6.54, 11.94)	5.21 (-1.13, 11.94)
Subtropical zone	2.32 (-0.29, 4.99)	-1.59 (-4.10, 0.98)	8.79 (6.60, 11.02)

Supplementary Table 6. Relative risk ratios (RRR) for effect estimates of heat wave at lag 0-1 days among two different subgroups.

Variable	Daytime only	Nighttime only	Compound
Cause			
Non-accidental mortality	1	1	1
Cardiovascular mortality	0.997(0.913,1.088)	1.037(0.957,1.125)	1.036(0.993,1.080)
IHD mortality	1.034(0.934,1.144)	1.063(0.957,1.18)	1.047(0.997,1.100)
Stroke mortality	0.97(0.883,1.065)	1.015(0.932,1.104)	1.027(0.977,1.079)
Respiratory mortality	1.09(0.985,1.206)	1.103(1.006,1.21)	1.068(1.012,1.127)
COPD mortality	1.09(0.980,1.212)	1.068(0.965,1.182)	1.060(1.000,1.123)
Gender			
Male	1	1	1
Female	0.991(0.908,1.081)	1.031(0.951,1.118)	1.017(0.973,1.063)
Age (years)			
0-74	1	1	1
75+	1.002(0.915,1.098)	1.133(1.046,1.228)	1.093(1.044,1.145)
Educational level			
Illiterate	1	1	1
Primary school or higher	0.97(0.884,1.065)	0.871(0.801,0.948)	0.937(0.892,0.984)
Region			
Urban	1	1	1
Rural	0.988(0.956,1.022)	1.096(1.054,1.14)	0.966(0.931,1.002)
Climate			
Mid-temperature zone	1	1	1
Warm temperature zone	0.978(0.935,1.023)	0.990(0.932,1.052)	1.027(0.962,1.097)
Qinghai-Tibet alpine zone	0.934(0.871,1.001)	0.983(0.886,1.091)	0.984(0.910,1.064)
Subtropical zone	0.969(0.926,1.014)	0.946(0.892,1.003)	1.018(0.967,1.072)

Note. IHD denotes ischemic heart disease; COPD denotes chronic obstructive pulmonary disease.

Supplementary Table 7. Percentage change in mortality risk during cold spell at daytime only, nighttime only and compound across lag 0-14 days by cause, individual characteristics, regions, and climate.

Variable	Daytime only	Nighttime only	Compound
Cause			
Non-accidental mortality	7.79 (1.71, 14.23)	11.94 (6.31, 17.86)	16.25 (13.11, 19.47)
Cardiovascular mortality	7.44 (0.63, 14.72)	16.12 (9.12, 23.57)	20.38 (16.57, 24.32)
IHD mortality	11.43 (2.51, 21.13)	18.97 (8.58, 30.34)	21.76 (16.89, 26.82)
Stroke mortality	4.56 (-2.85, 12.53)	13.57 (6.17, 21.48)	19.35 (14.54, 24.37)
Respiratory mortality	17.48 (8.15, 27.61)	23.49 (14.33, 33.38)	24.18 (18.53, 30.09)
COPD mortality	17.48 (7.48, 28.41)	19.56 (9.52, 30.51)	23.20 (17.09, 29.63)
Gender			
Male	8.30 (1.75, 15.28)	10.84 (5.35, 16.61)	15.17 (11.90, 18.52)
Female	7.30 (0.93, 14.07)	14.27 (7.30, 21.69)	17.15 (13.26, 21.17)
Age (years)			
0-74	7.46 (1.57, 13.69)	5.48 (0.16, 11.09)	11.20 (8.11, 14.37)
75+	7.69 (0.28, 15.65)	19.54 (12.44, 27.09)	21.56 (17.24, 26.05)
Educational level			
Illiterate	8.83 (1.42, 16.78)	21.00 (13.42, 29.09)	20.50 (15.91, 25.27)
Primary school or higher	5.61 (-0.59, 12.20)	5.45 (-0.01, 11.20)	12.92 (9.58, 16.37)
Region			
Urban	3.64 (-5.06, 13.14)	15.68 (8.25, 23.62)	15.25 (10.78, 19.89)
Rural	10.77 (2.54, 19.66)	9.67 (2.03, 17.87)	16.97 (12.66, 21.46)
Climate			
Mid-temperature zone	11.72 (-5.97, 32.73)	-11.43 (-24.92, 4.48)	8.53 (2.89, 14.49)
Warm temperature zone	-4.99 (-18.72, 11.05)	13.39 (2.29, 25.70)	14.24 (11.26, 17.30)
Qinghai-Tibet alpine zone	2.51 (-10.72, 17.70)	22.32 (4.24, 43.54)	12.13 (0.67, 24.89)
Subtropical zone	16.24 (8.50, 24.53)	15.93 (8.39, 24.00)	21.35 (15.88, 27.08)

Note. IHD denotes ischemic heart disease; COPD denotes chronic obstructive pulmonary disease.

Supplementary Table 8. Relative risk ratios (RRR) for effect estimates of cold spell at lag 0-14 days among two different subgroups.

Variable	Daytime only	Nighttime only	Compound
Cause			
Non-accidental mortality	1	1	1
Cardiovascular mortality	1.009(0.98,1.038)	0.999(0.968,1.031)	1.032(0.997,1.068)
IHD mortality	1.04(1.001,1.081)	1.012(0.970,1.055)	1.042(0.993,1.094)
Stroke mortality	0.996(0.963,1.030)	0.993(0.958,1.030)	1.022(0.985,1.060)
Respiratory mortality	1.000(0.960,1.041)	1.015(0.975,1.056)	1.016(0.974,1.060)
COPD mortality	1.002(0.96,1.045)	1.001(0.956,1.048)	1.001(0.955,1.049)
Gender			
Male	1	1	1
Female	1.023(0.993,1.053)	1.01(0.978,1.044)	1.033(0.999,1.069)
Age (years)			
0-74	1	1	1
75+	1.027(0.996,1.06)	0.997(0.966,1.03)	1.071(1.035,1.108)
Educational level			
Illiterate	1	1	1
Primary school or higher	0.989(0.959,1.02)	1.014(0.979,1.049)	0.940(0.907,0.975)
Region			
Urban	1	1	1
Rural	1.069(0.951,1.201)	0.948(0.86,1.046)	1.015(0.961,1.072)
Climate			
Mid-temperature zone	1	1	1
Warm temperature zone	0.85(0.675,1.072)	1.279(1.053,1.554)	1.053(0.992,1.117)
Qinghai-Tibet alpine zone	0.918(0.737,1.143)	1.381(1.097,1.738)	1.033(0.916,1.165)
Subtropical zone	1.04(0.866,1.25)	1.309(1.095,1.565)	1.118(1.042,1.200)

Note. IHD denotes ischemic heart disease; COPD denotes chronic obstructive pulmonary disease.

Supplementary Table 9. Percentage change in mortality risk during heat wave at daytime only, nighttime only and compound across lag 0 day by cause, individual characteristics, regions, and climate.

Variable	Daytime only	Nighttime only	Compound
Cause			
Non-accidental mortality	2.29 (0.94, 3.67)	1.60 (0.08, 3.13)	6.87 (5.15, 8.62)
Cardiovascular mortality	3.15 (1.16, 5.19)	1.96 (-0.16, 4.13)	9.14 (6.58, 11.77)
IHD mortality	5.23 (1.94, 8.63)	3.72 (0.46, 7.09)	9.99 (5.87, 14.27)
Stroke mortality	2.17 (-0.38, 4.79)	0.88 (-1.74, 3.58)	8.94 (6.17, 11.78)
Respiratory mortality	1.85 (-1.38, 5.19)	2.47 (-0.73, 5.77)	8.30 (4.58, 12.15)
COPD mortality	2.41 (-0.99, 5.92)	1.23 (-2.66, 5.27)	7.01 (2.88, 11.30)
Gender			
Male	1.55 (0.10, 3.02)	1.41 (-0.33, 3.17)	5.75 (3.96, 7.58)
Female	3.90 (1.88, 5.97)	1.96 (-0.11, 4.08)	8.18 (5.69, 10.72)
Age (years)			
0-74	1.83 (0.22, 3.47)	1.90 (0.21, 3.62)	3.98 (2.24, 5.75)
75+	3.35 (1.18, 5.57)	1.40 (-0.60, 3.44)	9.88 (7.30, 12.52)
Educational level			
Illiterate	3.77 (1.48, 6.10)	0.94 (-1.02, 2.93)	9.89 (6.98, 12.88)
Primary school or higher	2.34 (0.77, 3.94)	2.11 (0.17, 4.09)	5.35 (3.71, 7.01)
Region			
Urban	2.54 (0.22, 4.92)	2.12 (-0.34, 4.65)	8.35 (6.31, 10.43)
Rural	2.18 (0.52, 3.87)	1.24 (-0.66, 3.18)	5.79 (3.31, 8.33)
Climate			
Mid-temperature zone	4.48 (1.54, 7.50)	4.05 (-0.45, 8.75)	5.89 (1.57, 10.39)
Warm temperature zone	2.26 (-0.14, 4.71)	3.03 (0.64, 5.47)	6.88 (2.78, 11.13)
Qinghai-Tibet alpine zone	-1.93 (-7.08, 3.51)	1.92 (-4.16, 8.39)	5.40 (0.09, 10.98)
Subtropical zone	2.27 (0.14, 4.45)	-0.50 (-2.53, 1.57)	6.75 (4.85, 8.69)

Note. IHD denotes ischemic heart disease; COPD denotes chronic obstructive pulmonary disease.

Supplementary Table 10. Percentage change in mortality risk during cold spell at daytime only, nighttime only and compound across lag 0 day by cause, individual characteristics, regions, and climate.

Variable	Daytime only	Nighttime only	Compound
Cause			
Non-accidental mortality	1.54 (0.31, 2.78)	2.19 (0.92, 3.49)	1.43 (0.23, 2.65)
Cardiovascular mortality	2.35 (0.83, 3.90)	3.00 (1.38, 4.65)	2.93 (1.42, 4.46)
IHD mortality	4.65 (2.35, 7.01)	3.61 (1.30, 5.97)	3.89 (1.71, 6.11)
Stroke mortality	0.88 (-1.14, 2.95)	2.38 (0.06, 4.74)	2.94 (1.02, 4.91)
Respiratory mortality	1.06 (-1.02, 3.18)	3.27 (1.06, 5.54)	5.28 (2.88, 7.74)
COPD mortality	0.61 (-1.73, 3.00)	3.07 (0.57, 5.64)	5.18 (2.47, 7.96)
Gender			
Male	1.63 (0.26, 3.01)	1.91 (0.36, 3.48)	1.36 (0.05, 2.70)
Female	1.21 (-0.34, 2.79)	2.58 (1.01, 4.18)	1.74 (0.27, 3.24)
Age (years)			
0-74	1.29 (-0.04, 2.64)	1.53 (-0.07, 3.16)	-0.72 (-1.97, 0.55)
75+	1.58 (-0.04, 3.23)	2.78 (1.24, 4.35)	3.64 (2.09, 5.22)
Educational level			
Illiterate	2.22 (0.63, 3.84)	2.55 (1.04, 4.09)	2.63 (0.93, 4.35)
Primary school or higher	0.46 (-1.04, 1.98)	1.74 (0.14, 3.37)	0.59 (-0.80, 2.01)
Region			
Urban	0.02 (-1.90, 1.98)	2.91 (0.83, 5.03)	2.18 (0.42, 3.97)
Rural	2.59 (1.06, 4.16)	1.68 (0.06, 3.32)	0.86 (-0.79, 2.53)
Climate			
Mid-temperature zone	5.36 (2.14, 8.67)	-1.31 (-5.50, 3.08)	0.23 (-2.08, 2.59)
Warm temperature zone	-0.22 (-2.26, 1.87)	1.39 (-0.93, 3.76)	1.22 (-0.73, 3.21)
Qinghai-Tibet alpine zone	-0.99 (-4.87, 3.05)	5.92 (1.69, 10.32)	1.93 (-2.94, 7.05)
Subtropical zone	2.00 (0.23, 3.79)	2.66 (0.90, 4.46)	1.95 (0.03, 3.90)

Note. IHD denotes ischemic heart disease; COPD denotes chronic obstructive pulmonary disease.

Supplementary Table 11. Sensitivity analyses for effect estimates of heat wave on non-accidental mortality across lag 0-1 days by changing modelling strategies.

Model choice	Daytime only	Nighttime only	Compound
df for relative humidity: 3	2.95 (1.34, 4.58)	1.20 (-0.68, 3.12)	9.06 (7.08, 11.07)
df for relative humidity: 4	2.90 (1.28, 4.55)	1.13 (-0.75, 3.04)	9.02 (6.98, 11.09)
df for relative humidity: 5	2.82 (1.21, 4.46)	1.16 (-0.75, 3.10)	8.86 (6.82, 10.94)
df for air pressure: 3	2.82 (1.23, 4.43)	1.26 (-0.59, 3.14)	9.09 (7.06, 11.17)
df for air pressure: 4	2.79 (1.19, 4.40)	1.23 (-0.65, 3.15)	8.97 (6.90, 11.08)
df for air pressure: 5	2.82 (1.21, 4.46)	1.16 (-0.75, 3.10)	8.86 (6.82, 10.94)
Lag period: 8	2.90 (1.31, 4.52)	1.17 (-0.71, 3.08)	8.92 (6.87, 11.02)
Lag period: 10	2.82 (1.21, 4.46)	1.16 (-0.75, 3.10)	8.86 (6.82, 10.94)
Lag period: 14	3.08 (1.49, 4.69)	1.11 (-0.80, 3.04)	8.65 (6.60, 10.73)
Lag period: 21	2.95 (1.34, 4.59)	1.01 (-0.85, 2.90)	8.90 (6.84, 11.00)
df for per year: 4	2.82 (1.21, 4.46)	1.16 (-0.75, 3.10)	8.86 (6.82, 10.94)
df for per year: 6	2.82 (1.21, 4.46)	1.16 (-0.75, 3.10)	8.86 (6.82, 10.94)
df for per year: 8	2.82 (1.21, 4.46)	1.16 (-0.75, 3.10)	8.86 (6.82, 10.94)

Supplementary Table 12. Sensitivity analyses for effect estimates of cold spell on non-accidental mortality across lag 0-14 days by changing modelling strategies.

Model choice	Daytime only	Nighttime only	Compound
df for relative humidity: 3	8.34 (2.29, 14.74)	11.16 (5.61, 17.00)	16.22 (13.13, 19.41)
df for relative humidity: 4	7.91 (1.85, 14.34)	11.94 (6.30, 17.89)	16.09 (13.01, 19.25)
df for relative humidity: 5	7.79 (1.71, 14.23)	11.94 (6.31, 17.86)	16.25 (13.11, 19.47)
df for air pressure: 3	6.98 (1.03, 13.27)	11.49 (5.91, 17.36)	16.34 (13.18, 19.58)
df for air pressure: 4	7.40 (1.38, 13.78)	11.52 (5.87, 17.47)	16.46 (13.32, 19.69)
df for air pressure: 5	7.79 (1.71, 14.23)	11.94 (6.31, 17.86)	16.25 (13.11, 19.47)
Lag period: 8	7.62 (1.45, 14.17)	12.00 (6.56, 17.72)	16.08 (12.89, 19.36)
Lag period: 10	7.79 (1.71, 14.23)	11.94 (6.31, 17.86)	16.25 (13.11, 19.47)
Lag period: 14	5.92 (-0.29, 12.53)	11.83 (6.15, 17.80)	15.99 (12.74, 19.34)
Lag period: 21	5.00 (-1.02, 11.38)	10.04 (4.16, 16.26)	16.03 (12.77, 19.39)
df for per year: 4	7.79 (1.71, 14.23)	11.94 (6.31, 17.86)	16.25 (13.11, 19.47)
df for per year: 6	7.79 (1.71, 14.23)	11.94 (6.31, 17.86)	16.25 (13.11, 19.47)
df for per year: 8	7.79 (1.71, 14.23)	11.94 (6.31, 17.86)	16.25 (13.11, 19.47)

Supplementary Table 13. Percentage change in mortality risk during heat wave at daytime only, nighttime only and compound across lag 0-1 days by cause, individual characteristics, and regions.

Variable	Daytime only	Nighttime only	Compound
Cause			
Non-accidental mortality	2.93 (1.54, 4.35)	3.32 (1.96, 4.69)	9.58 (7.37, 11.85)
Cardiovascular mortality	3.33 (1.28, 5.43)	4.73 (2.77, 6.72)	13.50 (10.13, 16.98)
IHD mortality	2.28 (-0.99, 5.66)	6.93 (3.55, 10.42)	16.65 (11.38, 22.18)
Stroke mortality	3.89 (0.91, 6.96)	4.78 (2.15, 7.48)	12.30 (8.40, 16.33)
Respiratory mortality	4.63 (1.04, 8.35)	4.08 (0.68, 7.60)	13.19 (7.91, 18.73)
COPD mortality	3.47 (-0.70, 7.81)	3.21 (-0.94, 7.54)	13.02 (6.62, 19.79)
Gender			
Male	2.45 (0.69, 4.24)	3.59 (1.87, 5.33)	7.71 (5.29, 10.18)
Female	3.81 (1.56, 6.11)	2.98 (1.03, 4.96)	13.18 (10.09, 16.35)
Age (years)			
0-74	1.86 (0.03, 3.73)	2.81 (1.02, 4.63)	6.85 (4.66, 9.10)
≥75	4.44 (2.40, 6.52)	4.09 (2.20, 6.01)	13.52 (10.42, 16.70)
Educational level			
Illiterate	4.93 (2.69, 7.22)	3.55 (1.32, 5.84)	14.09 (10.49, 17.80)
Primary school or higher	1.95 (0.20, 3.72)	3.71 (1.93, 5.52)	7.81 (5.35, 10.32)
Region			
Urban	3.53 (1.36, 5.73)	4.33 (2.04, 6.66)	10.68 (7.47, 13.99)
Rural	2.49 (0.67, 4.36)	2.51 (0.72, 4.34)	8.79 (5.72, 11.95)
Climate			
Mid-temperature zone	4.02 (0.62, 7.54)	5.62 (1.87, 9.50)	10.74 (4.57, 17.28)
Warm temperature zone	3.02 (0.47, 5.63)	3.43 (1.08, 5.84)	13.02 (7.89, 18.40)
Qinghai-Tibet alpine zone	1.53 (-4.52, 7.96)	4.88 (-2.49, 12.80)	5.30 (-2.16, 13.32)
Subtropical zone	2.38 (0.13, 4.68)	2.15 (0.15, 4.20)	8.18 (5.93, 10.48)

Note. Heat wave is defined as at least two consecutive days with daily temperature measures (minimum temperature or maximum temperature) ≥ 90th percentile. IHD denotes ischemic heart disease; COPD denotes chronic obstructive pulmonary disease.

Supplementary Table 14. Percentage change in mortality risk during cold spell at daytime only, nighttime only and compound across lag 0-14 days by cause, individual characteristics, regions, and climate.

Variable	Daytime only	Nighttime only	Compound
Cause			
Non-accidental mortality	13.25 (7.93, 18.82)	15.55 (10.39, 20.94)	22.42 (18.16, 26.82)
Cardiovascular mortality	17.19 (10.82, 23.93)	20.08 (13.52, 27.03)	27.75 (22.40, 33.33)
IHD mortality	20.75 (12.10, 30.06)	21.41 (11.30, 32.45)	28.56 (21.26, 36.30)
Stroke mortality	13.75 (5.87, 22.21)	16.98 (9.15, 25.37)	27.91 (21.23, 34.96)
Respiratory mortality	22.07 (12.01, 33.04)	26.20 (16.15, 37.13)	30.86 (22.90, 39.34)
COPD mortality	22.83 (12.27, 34.38)	23.16 (11.98, 35.46)	29.15 (20.96, 37.90)
Gender			
Male	13.65 (7.42, 20.24)	14.45 (8.83, 20.36)	21.07 (16.61, 25.70)
Female	13.57 (7.89, 19.55)	16.72 (9.97, 23.89)	24.06 (18.61, 29.77)
Age (years)			
0-74	8.13 (2.43, 14.14)	9.15 (3.75, 14.84)	17.47 (13.17, 21.94)
75+	19.94 (13.44, 26.82)	22.07 (14.94, 29.64)	27.55 (21.89, 33.48)
Educational level			
Illiterate	14.29 (7.74, 21.25)	24.04 (15.88, 32.78)	29.06 (22.57, 35.88)
Primary school or higher	12.07 (5.78, 18.72)	9.57 (4.23, 15.20)	17.76 (13.26, 22.43)
Region			
Urban	10.07 (0.53, 20.51)	17.99 (11.63, 24.70)	22.41 (16.32, 28.83)
Rural	15.86 (9.68, 22.39)	14.05 (6.82, 21.76)	22.70 (16.80, 28.90)
Climate			
Mid-temperature zone	-8.88 (-24.06, 9.33)	0.01 (-12.15, 13.86)	15.59 (9.11, 22.46)
Warm temperature zone	9.49 (0.97, 18.73)	19.22 (9.86, 29.36)	22.25 (16.98, 27.75)
Qinghai-Tibet alpine zone	16.70 (-0.15, 36.39)	24.46 (8.12, 43.26)	14.17 (-5.81, 38.39)
Subtropical zone	22.48 (15.00, 30.45)	16.74 (9.19, 24.80)	27.70 (19.56, 36.39)

Note. Cold spell is defined as at least two consecutive days with daily temperature measures ≤ 10th percentile. IHD denotes ischemic heart disease; COPD denotes chronic obstructive pulmonary disease.

Supplementary Table 15. Relative risk ratios (RRR) for effect estimates of heat wave at lag 0-1 days between daytime-nighttime compound and daytime-/nighttime-only events.

Variable	Daytime only	Nighttime only	Compound
Cause			
Non-accidental mortality	0.945(0.922,0.968)	0.929(0.905,0.955)	1
Cardiovascular mortality	0.923(0.889,0.958)	0.899(0.865,0.935)	1
IHD mortality	0.943(0.891,0.998)	0.902(0.851,0.956)	1
Stroke mortality	0.921(0.882,0.961)	0.903(0.864,0.944)	1
Respiratory mortality	0.929(0.881,0.980)	0.928(0.881,0.977)	1
COPD mortality	0.945(0.891,1.002)	0.929(0.875,0.986)	1
Gender			
Male	0.957(0.932,0.982)	0.939(0.913,0.967)	1
Female	0.942(0.908,0.976)	0.919(0.885,0.953)	1
Age (years)			
0-74	0.968(0.943,0.994)	0.963(0.937,0.99)	1
75+	0.928(0.894,0.964)	0.897(0.864,0.931)	1
Educational level			
Illiterate	0.918(0.881,0.955)	0.885(0.85,0.921)	1
Primary school or higher	0.965(0.94,0.99)	0.954(0.925,0.983)	1
Region			
Urban	0.932(0.899,0.967	0.911(0.876,0.948)	1
Rural	0.592(0.516,0.679)	0.642(0.559,0.737)	1
Climate			
Mid-temperature zone	0.988(0.93,1.049)	0.973(0.907,1.044)	1
Warm temperature zone	0.94(0.893,0.991)	0.938(0.888,0.991)	1
Qinghai-Tibet alpine zone	0.937(0.86,1.021)	0.972(0.871,1.085)	1
Subtropical zone	0.941(0.910,0.972)	0.905(0.875,0.935)	1

Note. IHD denotes ischemic heart disease; COPD denotes chronic obstructive pulmonary disease.

Supplementary Table 16. Relative risk ratios (RRR) for effect estimates of cold spell at lag 0-14 days between daytime-nighttime compound and daytime-/nighttime-only events.

Variable	Daytime only	Nighttime only	Compound
Cause			
Non-accidental mortality	0.927(0.87,0.989)	0.963(0.908,1.021)	1
Cardiovascular mortality	0.893(0.830,0.960)	0.965(0.899,1.035)	1
IHD mortality	0.915(0.834,1.004)	0.977(0.884,1.080)	1
Stroke mortality	0.876(0.805,0.953)	0.952(0.879,1.03)	1
Respiratory mortality	0.946(0.860,1.040)	0.994(0.909,1.088)	1
COPD mortality	0.954(0.861,1.056)	0.970(0.877,1.074)	1
Gender			
Male	0.94(0.878,1.007)	0.962(0.908,1.020)	1
Female	0.916(0.854,0.982)	0.975(0.908,1.048)	1
Age (years)			
0-74	0.966(0.907,1.029)	0.949(0.894,1.006)	1
75+	0.886(0.818,0.96)	0.983(0.916,1.056)	1
Educational level			
Illiterate	0.903(0.833,0.979)	1.004(0.931,1.083)	1
Primary school or higher	0.935(0.874,1.001)	0.934(0.878,0.993)	1
Region			
Urban	0.899(0.817,0.99)	1.004(0.929,1.084)	1
Rural	0.947(0.869,1.032)	0.938(0.864,1.017)	1
Climate			
Mid-temperature zone	1.029(0.859,1.233)	0.816(0.686,0.971)	1
Warm temperature zone	0.832(0.710,0.974)	0.992(0.892,1.103)	1
Qinghai-Tibet alpine zone	0.914(0.767,1.089)	1.091(0.9000,1.323)	1
Subtropical zone	0.958(0.882,1.041)	0.955(0.881,1.037)	1

Note. IHD denotes ischemic heart disease; COPD denotes chronic obstructive pulmonary disease.

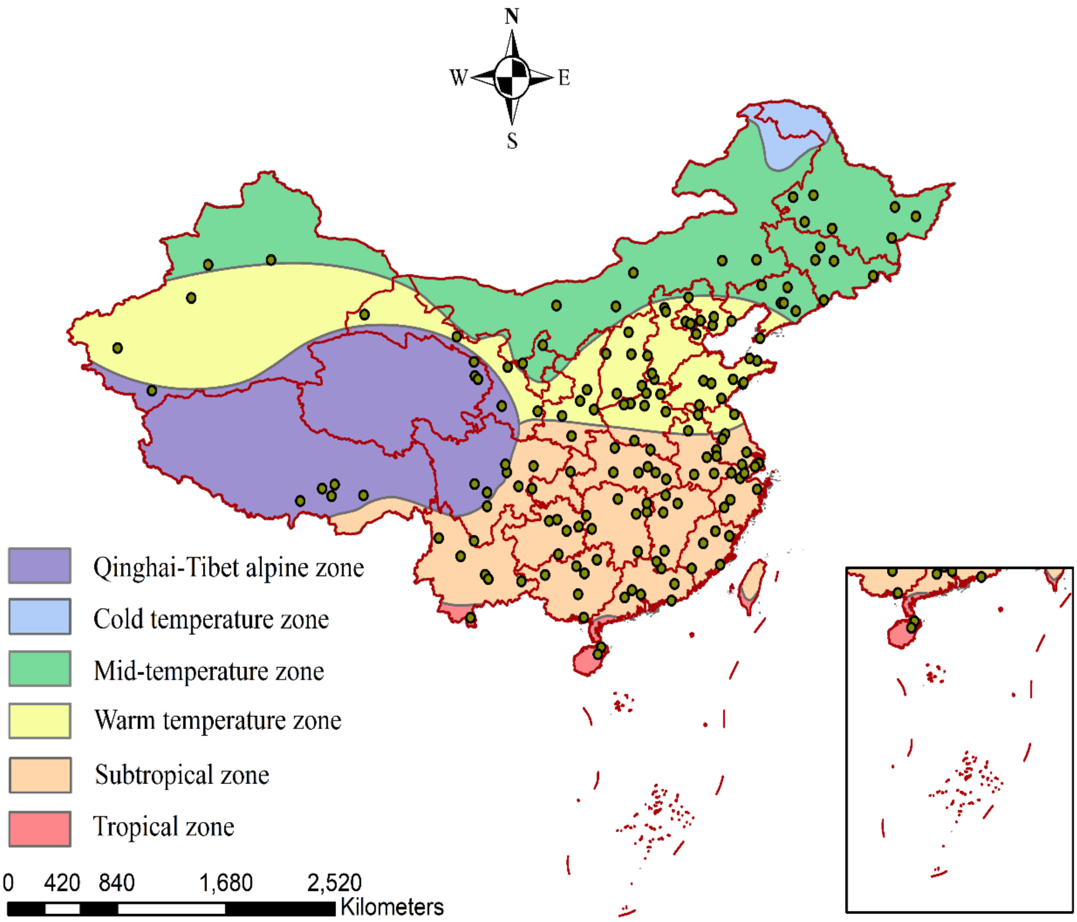

Supplementary Fig. 1. Geographical distribution of 161 Chinese communities, divided into six different temperature and geographic zones.

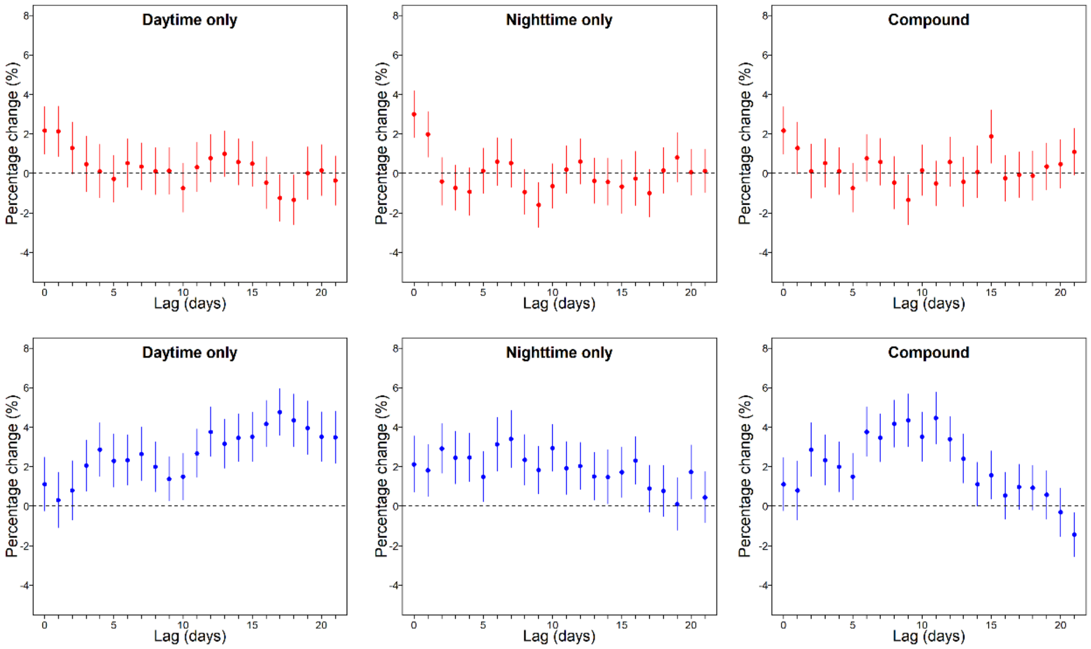

Supplementary Fig. 2. Lag patterns of heat wave (red lines) and cold spell (blue lines) on non-accidental mortality at daytime only, nighttime only and compound.

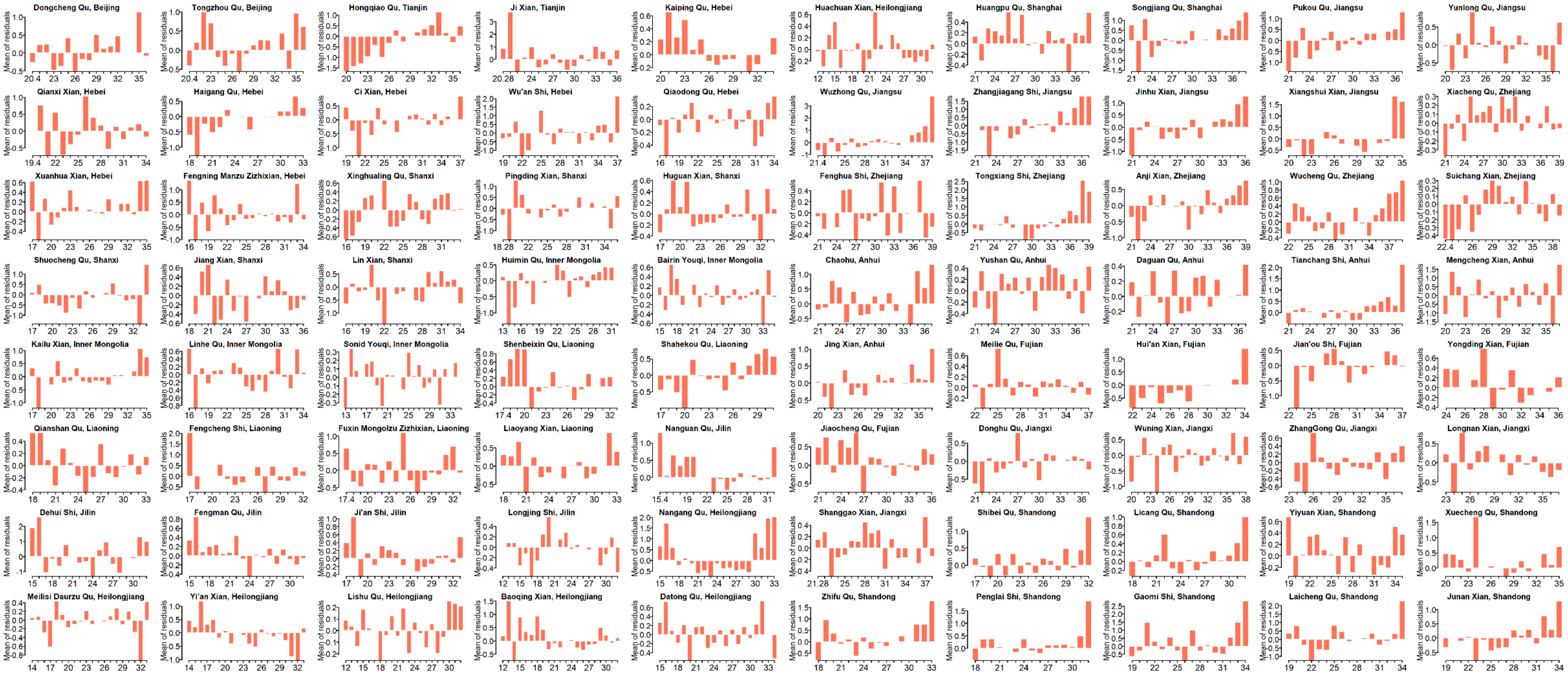

Supplementary Fig. 3. Barplots for determining heat wave definition on the basis of daily maximum temperature in the first set of Chinese communities.

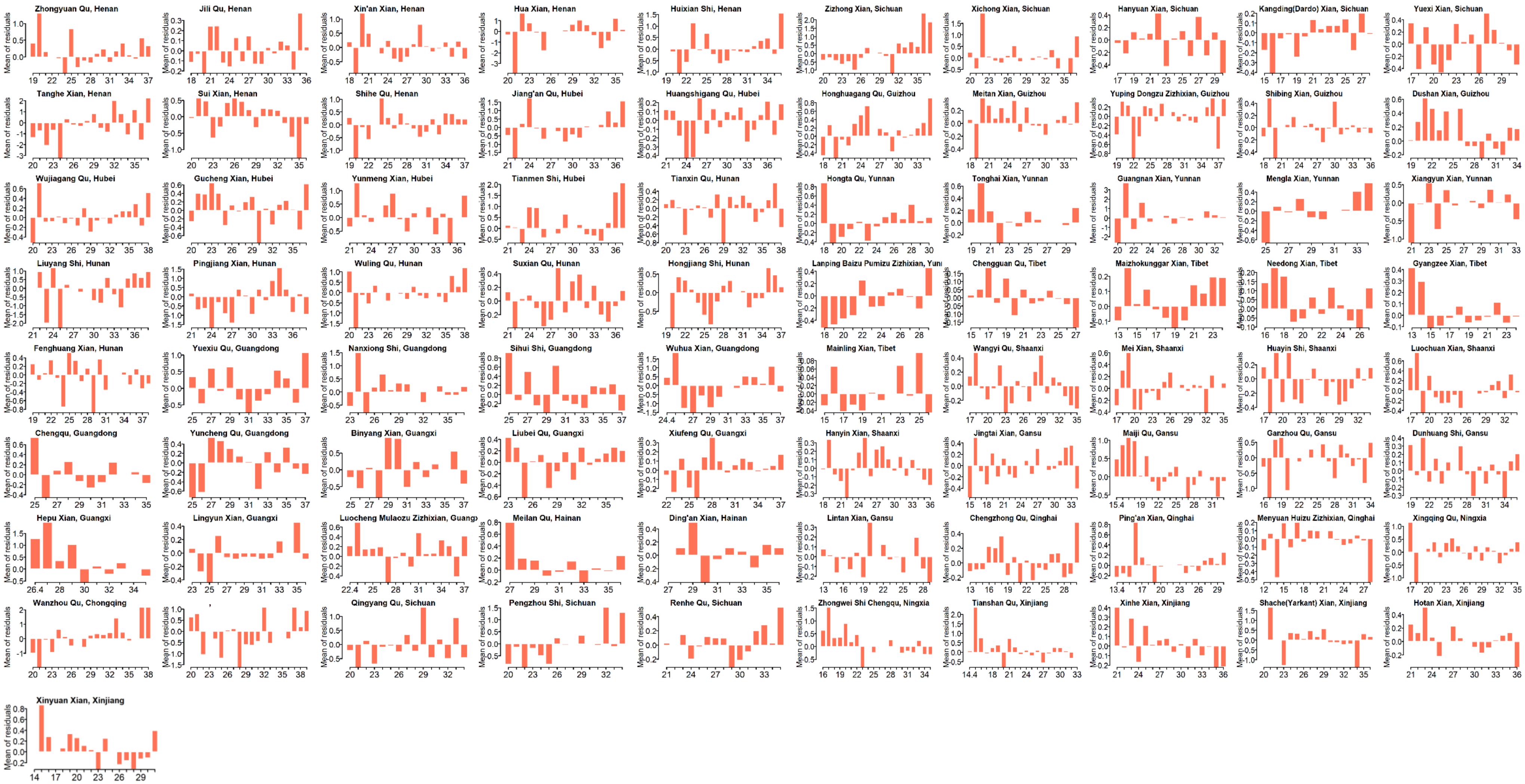

Supplementary Fig. 4. Barplots for determining heat wave definition on the basis of daily maximum temperature in the second set of Chinese communities.

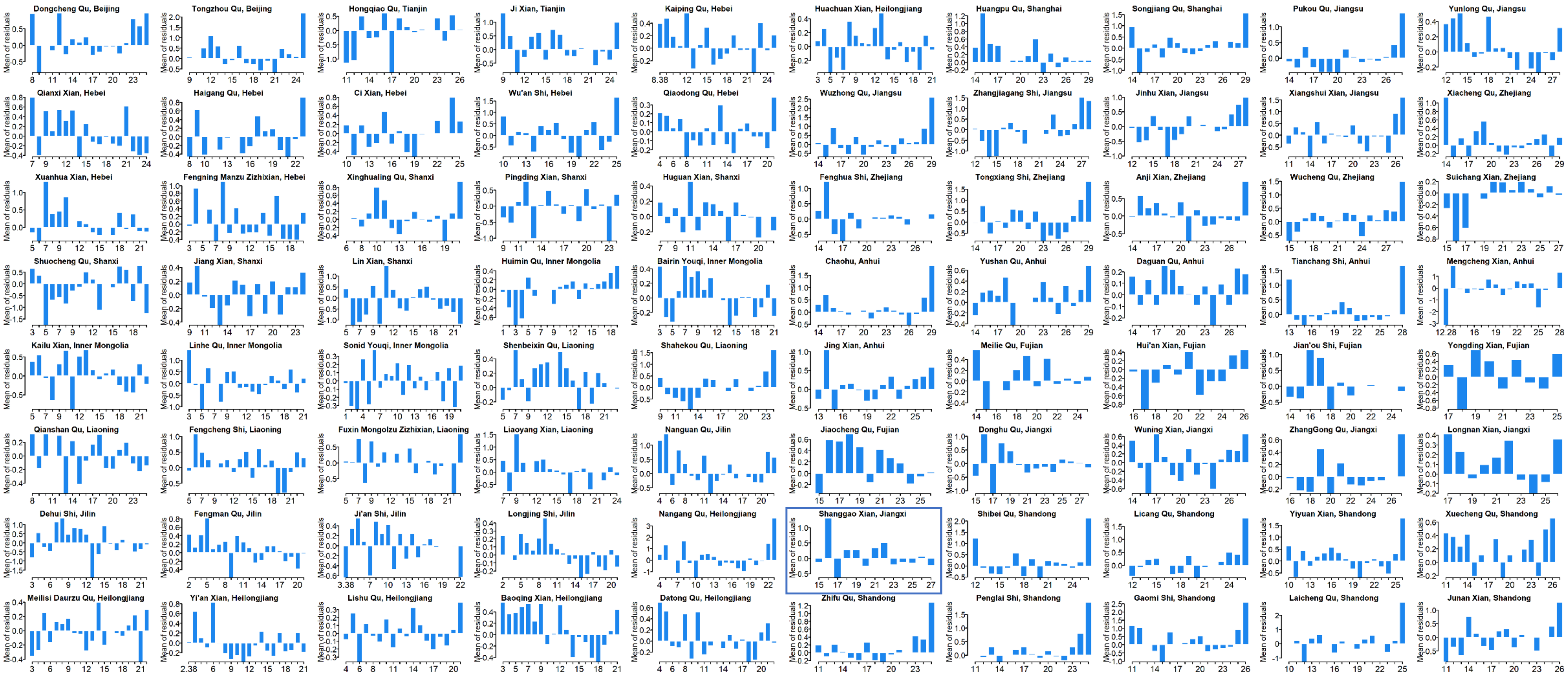

Supplementary Fig. 5. Barplots for determining heat wave definition on the basis of daily minimum temperature in the first set of Chinese communities.

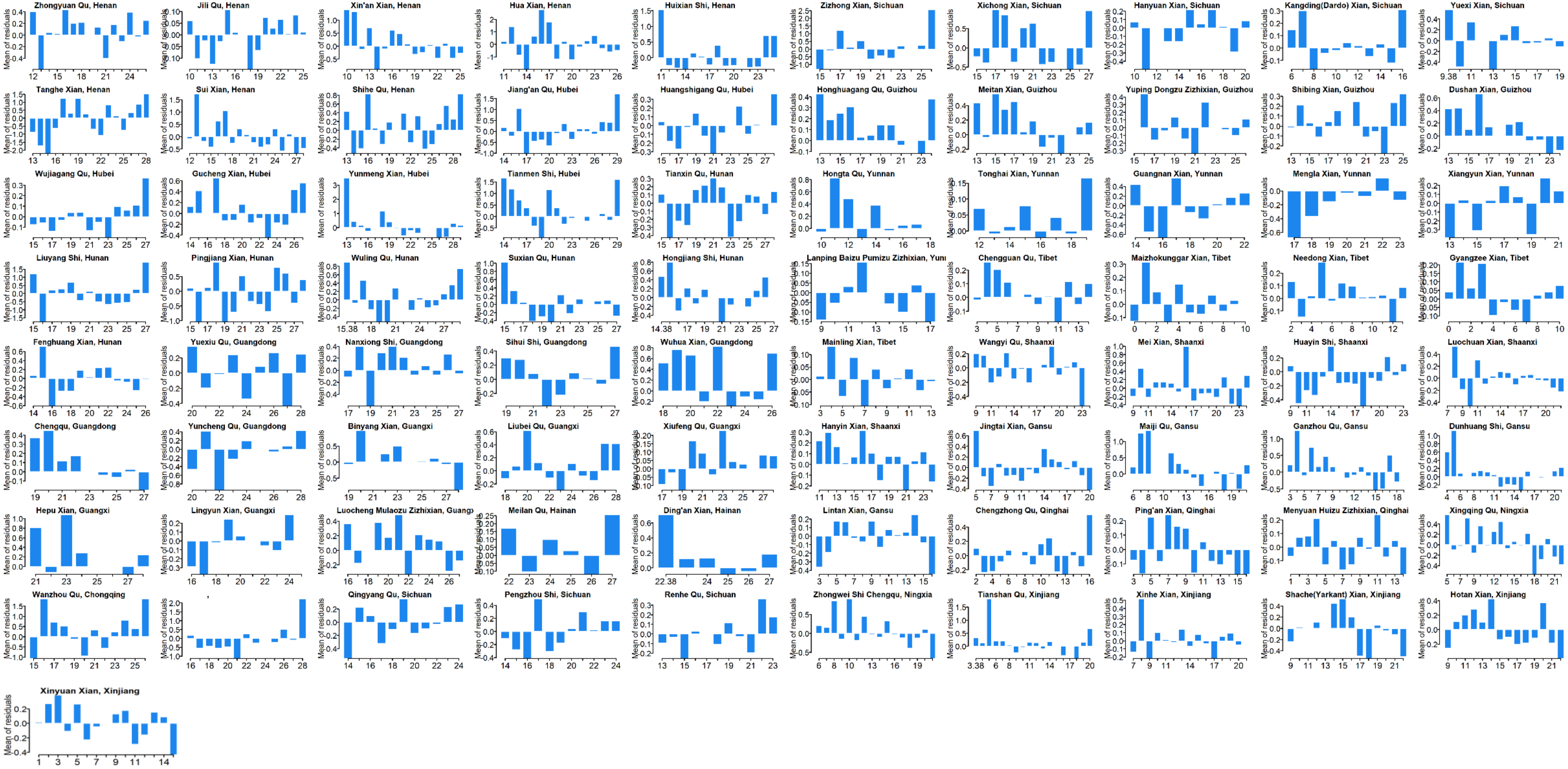

Supplementary Fig. 6. Barplots for determining heat wave definition on the basis of daily minimum temperature in the second set of Chinese communities.
