# Supplementary material for: Drivers of associations between daytime-nighttime compound temperature extremes and mortality in China
Source: Commun Med (Lond). 2024 Jun 27;4:125. doi: 10.1038/s43856-024-00557-0 (PMC11211425; doi:10.1038/s43856-024-00557-0)
Supplement: Supplementary file 3 — Description of Additional Supplementary Files [file 43856_2024_557_MOESM3_ESM.pdf]

Description of Additional Supplementary Files

File name: Supplementary Data 1

File Description: The information of disease surveillance points and the identified definitions of heat wave and cold spell for 161 Chinese communities during 2007-2013.

File name: Supplementary Data 2

File Description: Source data for the Figure 1

File name: Supplementary Data 3

File Description: Source data for the Figure 2
