# Supplementary material for: Drivers of associations between daytime-nighttime compound temperature extremes and mortality in China
Source: Commun Med (Lond). 2024 Jun 27;4:125. doi: 10.1038/s43856-024-00557-0 (PMC11211425; doi:10.1038/s43856-024-00557-0)
Supplement: Supplementary file 7 — Reporting Summary [file 43856_2024_557_MOESM7_ESM.pdf]

nature portfolio

Corresponding author(s): Jun Yang & Qiyong Liu

Last updated by author(s): Feb 15, 2024

Reporting Summary

Nature Portfolio wishes to improve the reproducibility of the work that we publish. This form provides structure for consistency and transparency in reporting. For further information on Nature Portfolio policies, see our Editorial Policies and the Editorial Policy Checklist.

Statistics

For all statistical analyses, confirm that the following items are present in the figure legend, table legend, main text, or Methods section.

n/a	Confirmed	
☒	☐	The exact sample size (*n*) for each experimental group/condition, given as a discrete number and unit of measurement
☒	☐	A statement on whether measurements were taken from distinct samples or whether the same sample was measured repeatedly
☐	☒	The statistical test(s) used AND whether they are one- or two-sided<br>*Only common tests should be described solely by name; describe more complex techniques in the Methods section.*
☐	☒	A description of all covariates tested
☒	☐	A description of any assumptions or corrections, such as tests of normality and adjustment for multiple comparisons
☐	☒	A full description of the statistical parameters including central tendency (e.g. means) or other basic estimates (e.g. regression coefficient) AND variation (e.g. standard deviation) or associated estimates of uncertainty (e.g. confidence intervals)
☐	☒	For null hypothesis testing, the test statistic (e.g. *F*, *t*, *r*) with confidence intervals, effect sizes, degrees of freedom and *P* value noted<br>*Give P values as exact values whenever suitable.*
☒	☐	For Bayesian analysis, information on the choice of priors and Markov chain Monte Carlo settings
☒	☐	For hierarchical and complex designs, identification of the appropriate level for tests and full reporting of outcomes
☐	☒	Estimates of effect sizes (e.g. Cohen's *d*, Pearson's *r*), indicating how they were calculated

Our web collection on statistics for biologists contains articles on many of the points above.

Software and code

Policy information about availability of computer code

Data collection	All the data preparations and analyses were conducted through the R Foundation for Statistical Computing, version 3.5.1.
Data analysis	All the data preparations and analyses were conducted through the R Foundation for Statistical Computing, version 3.5.1.

For manuscripts utilizing custom algorithms or software that are central to the research but not yet described in published literature, software must be made available to editors and reviewers. We strongly encourage code deposition in a community repository (e.g. GitHub). See the Nature Portfolio guidelines for submitting code & software for further information.

Data

Policy information about availability of data

All manuscripts must include a data availability statement. This statement should provide the following information, where applicable:

- Accession codes, unique identifiers, or web links for publicly available datasets
- A description of any restrictions on data availability
- For clinical datasets or third party data, please ensure that the statement adheres to our policy

The dataset generated and analyzed during this study are available (with some institutional limitations) from the corresponding authors upon reasonable request. The mortality data can be obtained from the Chinese Center for Disease Control and Prevention (China CDC) under the agreement to not engage in the unauthorized distribution of the raw data to a third party and to use the data for scientific research only (http://www.phsciencedata.cn/Share/en/index.jsp).

Historical temperature data are available at the dataset of daily climate data from Chinese surface stations of the China Meteorological Data Service Center (http://data.cma.cn/).

Research involving human participants, their data, or biological material

Policy information about studies with human participants or human data. See also policy information about sex, gender (identity/presentation), and sexual orientation and race, ethnicity and racism.

Reporting on sex and gender	Stratification analyses with the two-stage analytic approach were conducted by the level of humidity, cause, gender, age group, educational attainment and region.
Reporting on race, ethnicity, or other socially relevant groupings	Stratification analyses with the two-stage analytic approach were conducted by the level of humidity, cause, gender, age group, educational attainment and region.
Population characteristics	The ICD-10 (International Classification of Diseases, 10th version) was applied to classify the causes of death.
Recruitment	Observational study so no recruitment
Ethics oversight	This study involved only secondary analysis of daily aggregated and de-identified data, and is classified as exempt from IRB approval in the Chinese legal documents on ethics review that was issued by the National Health Commission of the People's Republic of China.

Note that full information on the approval of the study protocol must also be provided in the manuscript.

Field-specific reporting

Please select the one below that is the best fit for your research. If you are not sure, read the appropriate sections before making your selection.

☐ Life sciences ☐ Behavioural & social sciences ☒ Ecological, evolutionary & environmental sciences

For a reference copy of the document with all sections, see nature.com/documents/nr-reporting-summary-flat.pdf

Ecological, evolutionary & environmental sciences study design

All studies must disclose on these points even when the disclosure is negative.

Study description	Time-series study
Research sample	2,742,717 non-accidental deaths, 1,274,558 cardiovascular deaths, and 397,738 respiratory deaths
Sampling strategy	The daily mortality data in 161 communities (64 city districts in urban areas and 97 counties in rural areas) were collected from the nationwide Disease Surveillance Points system (DSPs), which was established using the multi-stage random sampling strategy.
Data collection	Daily mortality in 161 communities were collected from Chinese Center for Disease Control and Prevention; daily temperature data (minimum, maximum and mean temperatures) from 839 weather stations during 2007-2013 were firstly collected from the China Meteorological Data Service Center; community-level demographic data in 2010 from the China's 2010 population census, along with gross domestic product (GDP) per capita from the city-level or county-level statistical yearbook; normalized difference vegetation index (NDVI) data from the Moderate Resolution Imaging Spectroradiometer NASA's Earth Observing System
Timing and spatial scale	Daily mortality were obtained from 161 communities during 2007-2013; daily temperature during 2007-2013 were interpolated separately into 1km×1km; demographic data were at community-level; NDVI at 1-km resolution; PM2.5 at 36 km×36 km resolution
Data exclusions	No data were excluded from the analyses.
Reproducibility	This study is time-series design. Reproducibility can be tested using similar model specifications and statistical indicator in this study.
Randomization	This study is time-series design. Randomization was not applicable.
Blinding	This study is time-series design. Blinding was not applicable.

Did the study involve field work? ☐ Yes ☒ No

Reporting for specific materials, systems and methods

We require information from authors about some types of materials, experimental systems and methods used in many studies. Here, indicate whether each material, system or method listed is relevant to your study. If you are not sure if a list item applies to your research, read the appropriate section before selecting a response.

Materials & experimental systems

n/a	Involved in the study
☒	☐ Antibodies
☒	☐ Eukaryotic cell lines
☒	☐ Palaeontology and archaeology
☒	☐ Animals and other organisms
☒	☐ Clinical data
☒	☐ Dual use research of concern
☒	☐ Plants

Methods

n/a	Involved in the study
☒	☐ ChIP-seq
☒	☐ Flow cytometry
☒	☐ MRI-based neuroimaging

Plants

Seed stocks	*Report on the source of all seed stocks or other plant material used. If applicable, state the seed stock centre and catalogue number. If plant specimens were collected from the field, describe the collection location, date and sampling procedures.*
Novel plant genotypes	*Describe the methods by which all novel plant genotypes were produced. This includes those generated by transgenic approaches, gene editing, chemical/radiation-based mutagenesis and hybridization. For transgenic lines, describe the transformation method, the number of independent lines analyzed and the generation upon which experiments were performed. For gene-edited lines, describe the editor used, the endogenous sequence targeted for editing, the targeting guide RNA sequence (if applicable) and how the editor was applied.*
Authentication	*Describe any authentication procedures for each seed stock used or novel genotype generated. Describe any experiments used to assess the effect of a mutation and, where applicable, how potential secondary effects (e.g. second site T-DNA insertions, mosiacism, off-target gene editing) were examined.*
